# The Impact of Furfural on the Quality of Meads

**DOI:** 10.3390/molecules29010029

**Published:** 2023-12-20

**Authors:** Paweł Sroka, Tomasz Tarko, Aleksandra Duda

**Affiliations:** Department of Fermentation Technology and Microbiology, University of Agriculture in Krakow, ul. Balicka 122, 30-149 Kraków, Poland; aleksandra.duda@urk.edu.pl

**Keywords:** mead, honey wine, fermentation, furfural, furfuryl alcohol, *Saccharomyces cerevisiae*

## Abstract

Furfural is a naturally occurring compound in bee honey, classified as a fermentation inhibitor. The aim of this study was to ascertain the concentration of furfural in mead worts, prepared at room temperature (unsaturated) and heated to boiling for 10 to 70 min (saturated), with an extract of 25 to 45°Brix. Moreover, the impact of the furfural on the fermentation course of mead wort was assessed. For this purpose, fermentation tests were conducted using mead wort (30°Brix) to which furfural was added at concentrations ranging from 1 to 100 mg/L. HS-SPME-GC-TOF-MS analysis revealed that the furfural concentration in mead worts varied between 2.3 and 5.3 mg/L. In saturated worts, the concentration increased by 2.8 to 4.5 times. Acidification of mead wort prior to boiling led to further increase in furfural concentration. The greatest changes occurred in the least concentrated worts, having the lowest buffer capacity. The addition of furfural to the mead wort did not inhibit fermentation, and an increase in attenuation was observed in the samples containing 2 mg/L of furfural compared to the control. Throughout the fermentation most of the furfural was reduced to furfuryl alcohol.

## 1. Introduction

Mead is a fermented beverage with an ethanol content ranging from 8% to 18% *v*/*v* produced through the fermentation of mead wort obtained from diluted bee honey [1]. Depending on the ratio of water to honey in the mead wort, the drinks are called: półtorak (1:0.5), dwójniak (1:1), trójniak (1:2) and czwórniak (1:3). These names have been registered as Traditional Specialities Guaranteed (TSGs) and are protected throughout the European Union to preserve traditional production methods and recipes [2].

Mead worts are characterized by a high sugar content, which is one of the factors that inhibit the fermentation process. The high osmotic pressure resulting from the high concentration of the extract contributes to the relatively long adaptation time of the yeast [3]. This increases the synthesis of acetic acid [4,5]. Bee honey is characterized by low acidity and low content of substances necessary for yeast metabolism. Mead wort is usually additionally acidified with citric acid. It is also supplemented with salts containing ammonium ions, phosphates(V) and complex preparations based on yeast extracts [6,7,8,9,10]. A deficiency of compounds containing elements such as calcium, magnesium, phosphorus, nitrogen and vitamins, mainly from the B group, can lead to stuck and slow fermentation [1,7,11]. Acidification of mead worts is aimed at lowering the pH of the solution, reducing the risk of development of harmful microbiota that may lead to spoilage of the product, and balancing the sweet taste resulting from the high concentration of carbohydrates [6,12].

Problems with low attenuation of mead wort are also caused by the presence of compounds with antimicrobial activity in bee honey [13]. This group includes polyphenolic compounds, essential oils and amphiphiles, i.e., compounds with both hydrophilic and hydrophobic properties. The latter group includes medium molecular weight aliphatic acids, e.g., hexanoic, octanoic and decanoic acids, which in high concentrations and at low pH can contribute to fermentation inhibition [6,14]. These compounds have surface-active properties and are absorbed by suspensions and colloids present in the fermentation medium [15,16]. In the case of mead wort, yeast hulls, pollen, water-insoluble polymers and hydrocolloids can be added to accelerate fermentation. A similar effect can be achieved by immobilizing yeast cells on organic or inorganic supports [15,16,17,18,19,20].

According to the method of wort preparation, meads can be divided into saturated and unsaturated. Saturated mead is made from wort that is heated to boiling before fermentation [21]. Unsaturated meads are richer in aromatic substances of raw material origin, and partially retain the enzymatic activity of honey, which results in the formation of hydrogen peroxide and gluconolactone in the worts [22]. As a result of lactone hydrolysis, gluconic acid is produced, which lowers the pH of the wort environment. Increasing acidity increases the effective concentration of undissociated medium molecular weight fatty acids. These compounds are classified as fermentation inhibitors, which can also negatively affect the fermentation rate and significantly reduce the degree of sugar attenuation [23].

When saturated meads are obtained, microorganisms and enzymes present in mead wort are deactivated by heating, while proteins coagulate and some polyphenolic compounds and waxes are precipitated [3,6]. Saturated worts are characterized by faster fermentation and clarification and higher microbiological stability [24]. Heating mead worts above 70 °C deactivates glucose oxidase [22]. During heating, valuable aromatic compounds characteristic for the type of bee honey are evaporated, but also new substances are formed, which significantly affect the aroma [25]. Maillard reactions occur in heated worts, contributing to increased concentration of furan derivatives, including 5-hydroxymethylfurfural (HMF) and furfural [24]. These compounds occur naturally in bee honey, especially when stored at elevated temperatures [26]. HMF is formed by the dehydration of hexoses in an acidic environment, while furfural is formed by the dehydration of pentoses and the decomposition of ascorbic acid [27]. These reactions also take place during the preparation and fermentation of mead wort. The rate of formation of furan derivatives depends mainly on the substrate concentration, temperature and pH [26]. The concentrations of the mentioned substances depend on the type and storage conditions of the honey and are inversely proportional to the freshness of the product. At the same time, the method of mead wort production can significantly affect the concentration of furan derivatives in the fermented liquids [24].

Furfural is classified as a fermentation inhibitor, i.e., a factor that inhibits the fermentation process and reduces the attenuation of sugars in the wort. Furan compounds inhibit the growth of *S. cerevisiae* yeast and reduce the concentration of ethanol in the final products [27,28,29]. Furfural inhibits glycolysis and the fermentation rate, especially alcohol dehydrogenase, aldehyde dehydrogenase and pyruvate dehydrogenase [30,31,32,33]. In a study by Palmqvist et al. [34], a negative effect of a mixture of furfural and acetic acid on biomass growth and ethyl alcohol production was found. Allen et al. [35] showed that under aerobic conditions, the presence of furfural inhibits the growth of yeast, despite the presence of sufficient amounts of appropriate nutrients. Under anaerobic conditions, yeast cells reduce furfural to furfuryl alcohol [27]. The degree of furfural reduction is proportional to the inoculum density [34]. The furfural reducing agent is NADH, and the reaction leads to a reduction in the furfural concentration in the solution within a few to several dozen hours [36].

After inoculation of the mead worts with *S. cerevisiae* yeast, alcoholic fermentation lasts from several days to up to three months [1]. Once the fermentation process is complete, young meads are aged for several months to several years. To shorten the fermentation time of mead wort, one strategy is to select appropriate yeast strains that are adapted to the specific high-sugar environment [37]. Research was conducted on the adaptation of yeast cells to high sugar concentration solutions containing substances classified as fermentation inhibitors. The data showed that adaptation to a furfural-enriched environment led to a significant reduction in fermentation time. The adapted strains also showed an increased ability to degrade HMF and a significant increase in the conversion of furfural to furfuryl alcohol [38].

During the fermentation of mead worts, a synergistic effect is often observed as a result of high osmotic pressure, high ethanol concentration, and numerous substances classified as fermentation inhibitors on yeast cells. The objectives of this work were: (i) to evaluate the quantity of furfural produced during the preparation and saturation of mead worts and (ii) to determine the impact of various concentrations of furfural on the fermentation course and the parameters of young honey. Additionally, the amount of furfuryl alcohol generated in the young meads was monitored.

## 2. Results

### 2.1. The Influence of Heating Mead Wort on the Furfural Formation

**Figure 1:**
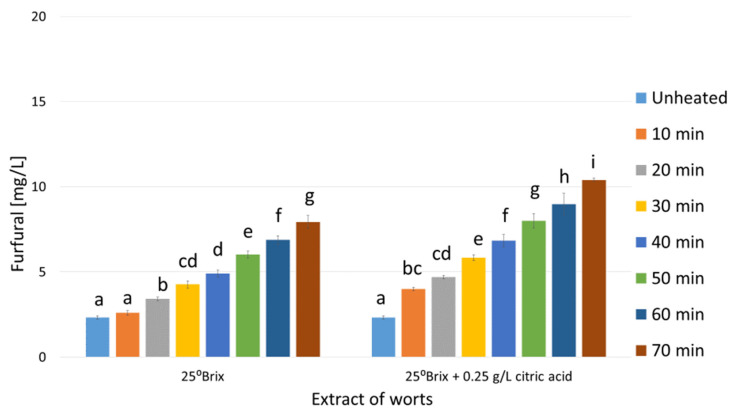
Changes in the concentration of furfural in mead wort with an extract of 25°Brix as a function of acidification and time of boiling. Values are expressed as the mean ± standard deviation. Means with different letters (a–i) are statistically different (*p* < 0.05).

**Figure 2:**
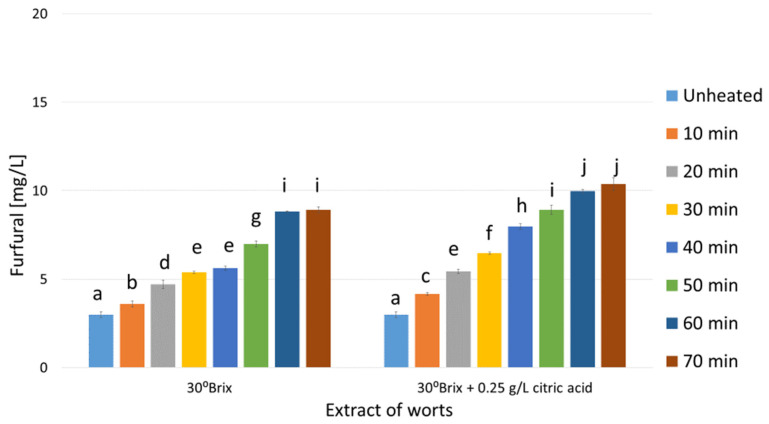
Changes in the concentration of furfural in mead wort with an extract of 30°Brix as a function of acidification and time of boiling. Values are expressed as the mean ± standard deviation. Means with different letters (a–j) are statistically different (*p* < 0.05).

**Figure 3:**
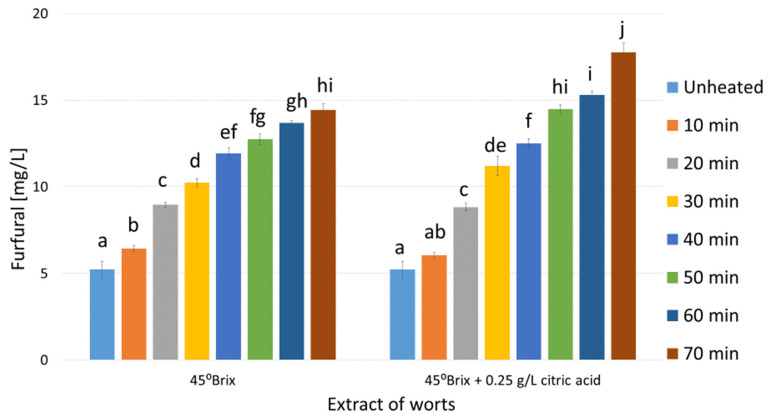
Changes in the concentration of furfural in mead wort with an extract of 45°Brix as a function of acidification and time of boiling. Values are expressed as the mean ± standard deviation. Means with different letters (a–j) are statistically different (*p* < 0.05).

As shown in Figure 1, Figure 2 and Figure 3 the unheated control worts contained furfural at concentrations ranging from 2.3 (mead wort with an extract of 25°Brix, Figure 1) to 5.2 mg/L (45°Brix, Figure 3). The differences in furfural concentrations resulted from the different amount of bee honey mixed with water, i.e., the dilution of the wort, which ranged approximately from two (1:1 in dwójniak mead, 45°Brix) to four (1:3 in czwórniak, 25°Brix). In heated mead worts, an increase in furfural concentration proportional to the heating time was observed (Figure 1, Figure 2 and Figure 3). In more diluted samples the percentage increase in furfural was higher. In the wort of unacidified czwórniak (25°Brix), the concentration of furfural increased by 242% after 70 min of heating compared to the unheated wort (Figure 1). In the acidified worts heated for 70 min, the concentration, compared to the corresponding unacidified worts, was even higher by between 14% (30°Brix, Figure 2) and 30% (25°Brix, Figure 1).

Since dwójniak mead, with the highest extract of 45°Brix, is most often produced from wort with an extract of 30°Brix by adding honey in portions during fermentation, further experiments were conducted only on trójniak mead worts with an extract of 30°Brix.

### 2.2. Fermentation of Mead Worts Supplemented with Furfural

**Figure 4:**
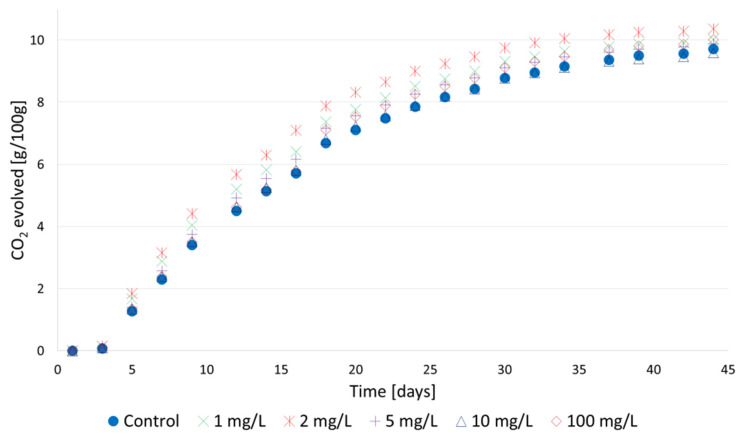
Carbon dioxide (g/100 g) released during the fermentation of mead worts without (control) and with furfural addition (1 to 100 mg/L).

During the fermentation of mead worts (30°Brix) with different concentrations of furfural (ranging from 1 to 100 mg/L), the weight of the samples was monitored. The loss of weight was due to the release of carbon dioxide produced during fermentation (Figure 4). During the first few days of the process, no changes were observed as a result of yeast adaptation to a high-sugar environment. Samples supplemented with furfural (from 1 to 5 mg/L) were generally characterized by a greater CO_2_ release as early as the fifth day of the process, proving a faster adaptation of yeast cells in the supplemented wort. After about one week, all samples were fermenting vigorously, with intense foaming and carbon dioxide release. In the third week, the violent fermentation ended and the post-fermentation period, characterized by low carbon dioxide release, began. The greatest CO_2_ release was found in samples supplemented with furfural at a concentration of 2 mg/L. The experiment indicates that even relatively high concentrations of furfural (100 mg/L) do not inhibit the fermentation of mead wort.

### 2.3. Young Meads’ Parameters

**Figure 5:**
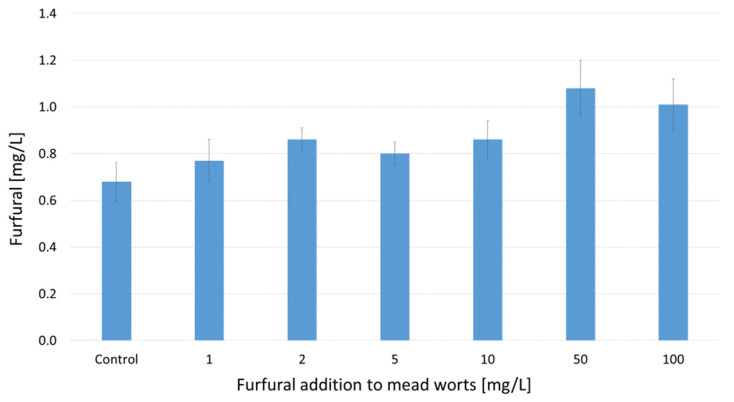
The concentrations of furfural in young meads determined after 7 weeks of fermentation of 30°Brix mead worts, with furfural added at concentrations ranging from 1 to 100 mg/L. Values are expressed as the mean ± standard deviation. The differences were not statistically significant.

**Figure 6:**
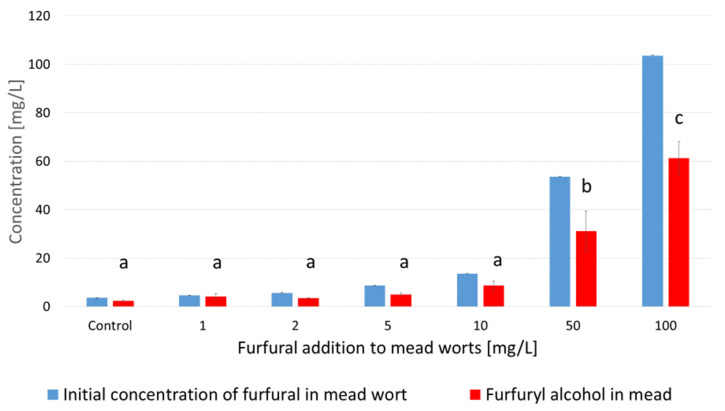
Initial concentrations of furfural in mead worts before fermentation and furfuryl alcohol concentrations in young meads, for samples with starting extracts of 30°Brix with furfural addition before fermentation at concentrations from 1 to 100 mg/L. Values are expressed as the mean ± standard deviation. Means with different letters (a–c) are statistically different (*p* < 0.05).

After 7 weeks of fermentation, young meads contained only 0.68 to 1.08 mg/L furfural (Figure 5), which means that the vast majority of the initial furfural content was decomposed during fermentation. The main product of furfural transformations was furfuryl alcohol (Figure 6). Its concentration in the fermented worts ranged from 2.3 to 61.3 mg/L, depending on the dose of furfural added. In samples supplemented with furfural at concentrations higher than 50 mg/L, more than 70% of this compound was reduced to furfuryl alcohol (Figure 6).

The ethanol concentration in the fermented samples ranged from 13.0 to 14.3% *v*/*v* (Table 1), and only samples with 2 mg/L furfural addition had higher ethanol concentrations after fermentation compared to the control sample. In this case, a slightly (more than 1% *v*/*v*) higher ethanol concentration was obtained. The alcohol degree obtained in the fermented samples was correlated with the amount of carbon dioxide released during fermentation (Figure 4).

The total extract content ranged from 146 to 163 g/L in young mead (Table 1), and no significant differences were found among the samples analyzed. Based on the results obtained, it can be concluded that furfural has no significant effect on the total acidity and volatile acidity of young meads. The total acidity ranged from 3.4 to 3.7 g/L, which is relatively low and the resulting meads require additional correction after maturation with the addition of an appropriate amount of citric acid. The pH during fermentation is particularly important for the proper course of the process. A low pH prevents the development of undesirable microbiota. Wine yeast is adapted to low pH, but values below 3.0 can slow down or inhibit fermentation. In the fermented mead worts, the pH was between 3.3 and 3.5. Fermentation of high-sugar mixtures contributes to osmotic stress acting on yeast cells, which under these conditions increase the synthesis of acetic acid, which is the main compound influencing volatile acidity. The volatile acidity values of the fermented samples did not differ significantly and ranged from 1.2 to 1.4 g/L, which was relatively high.

## 3. Discussion

As Figure 1, Figure 2 and Figure 3 display, heating mead worts significantly changes the contents of furfural. As a result of the Maillard reaction, furan derivatives are formed [24]. The conducted experiments showed that boiling the worts for 70 min increases the concentration of furfural. The growth was between 2.8 times in the 45°Brix wort (Figure 3) and about 4.5 times in the acidified 25°Brix wort sample (Figure 1). Unacidified worts contained between 7.9 and 14.4 mg/L of furfural (for 25°Brix and 45°Brix boiled worts, respectively), while the addition of citric acid before heating contributed to the increase in furfural concentration to values between 10.3 (25°Brix) and 17.7 mg/L (45°Brix), depending on the wort extract (Figure 1, Figure 2 and Figure 3). The relatively large impact of wort acidification on the concentration of furfural formed during heating is probably due to the low buffer capacity of the solutions and the lower pH of the worts, which catalyzes the reaction of formation of furan compounds [26].

It has been reported that furfural inhibits alcohol production by *Saccharomyces cerevisiae* in solutions containing 10 mg/L of this compound [27]. Previous studies have shown that furfural at a concentration of 4 g/L inhibits the yeast cell growth by 80% and ethanol production by 97% [30]. In our experiments, unheated wort contained from 2.3 (25°Brix) to 5.2 mg/L (45°Brix) of furfural, which should not significantly affect the fermentation process. However, boiled worts contained furfural at concentrations up to 18 mg/L, which, according to research [27], should significantly reduce the concentration of ethanol produced. In the case of fermentation of concentrated worts, the yeast is additionally subjected to osmotic stress, which may result in an increased inhibitory effect on the fermentation process.

Knowing the concentration of furfural produced during the heating of mead worts (Figure 1, Figure 2 and Figure 3), trójniak worts with an extract of 30°Brix supplemented with furfural were prepared in order to determine its effect on the fermentation process. However, our experiments showed that even high (over 100 mg/L) concentrations of furfural did not inhibit alcohol fermentation (Figure 4), and low concentrations (2 mg/L) of added furfural contributed to increased alcohol production (Table 1), probably as a result of better adaptation of cells to the environment [38].

Furfural inhibits the fermentation process and also significantly inhibits the growth of yeast [33]. It should be noted, that relatively large amounts of yeast are added during mead production, usually twice as much as in the case of fermentation of wine musts. Therefore, inhibition of yeast multiplication by furfural during the adaptation of mead worts may not be as important as in the case of wine production. In addition, the reduction of furfural to furfuryl alcohol occurs within a few tens of hours after fermentation initiation [34,39,40,41]. A relatively large number of yeast cells and an extended adaptation time of honey worts, up to several days [16], contribute to an increase in the rate of reduction of furfural to furfuryl alcohol. The obtained experimental results could be influenced by two factors: the relatively rapid reduction of furfural to furfuryl alcohol and the absence of an inhibitory effect of this compound on the concentration of ethyl alcohol in the turbulent phase of fermentation, which commenced in the second week following the addition of yeast to the wort (Figure 4).

*S. cerevisiae* cells rapidly decompose the furfural in the solution, yielding furfuryl alcohol as the main product (Figure 6). The reaction employs the reduced form of nicotinamide adenine dinucleotide (NADH) as the reducing agent, with the catalytic participation of alcohol dehydrogenase (ADH) [41].

Furfural + NADH + H^+^ = furfuryl alcohol + NAD^+^

A small portion of furfural may have been converted into 1-(2-furyl)-1-hydroxy-2-propanone and 1-(2-furyl)-propane-1,2-diol [42] or oxidized into furoic acid [33,39,41]. Furfural can also react with amino acids present in mead wort and produce colored compounds. According to Murata et al. [43], furfural reacts with lysine to generate the yellow dye furpipatide. All these processes lead to the final concentration of furfural in the fermented worts not exceeding 1.1 mg/L (Figure 5).

It has been demonstrated that adding a low concentration of furfural (2 mg/L) can speed up fermentation and increase the ethanol concentration by 1% *v*/*v* in the fermented samples (Table 1). This suggests that low furfural concentrations have a beneficial effect on yeast adaptation processes or cell metabolism aimed at reducing furfural to furfuryl alcohol [38].

Fermenting solutions with high initial wort extracts results in strong osmotic stress. Yeasts respond to high sugar concentrations by producing more acetic acid, as a result of interference with the reduction of acetaldehyde [5]. During the production of mead, significant amounts of acetic and succinic acids are produced. An increase in acidity lowers the pH and, in extreme cases, may contribute to the inhibition of fermentation [44]. Volatile acidity was high in all of the analyzed samples (Table 1). Nonetheless, the addition of furfural did not result in any significant changes in volatile acidity. This can be attributed to the rapid decomposition of furfural, most likely occurring during the yeast adaptation phase.

## 4. Materials and Methods

### 4.1. Biological Material

The mead worts were prepared from buckwheat honey (Sądecki Bartnik, Stróże, Poland) by appropriate dilution with potable water (96.0 mg/L Ca^2+^, 11.0 mg/L Mg^2+^, 37.4 mg/L Cl^−^, 49.0 mg/L SO_4_^2−^, pH 7.7).

Commercial wine yeast *Saccharomyces cerevisiae* Vintage White from Enartis (Navarrete, Spain) was used in the fermentation experiments. To prepare the yeast for fermentation, a yeast slurry was created by adding 10 g of dry yeast to 100 mL of sterile tap water. The resulting suspension, known as starter yeast, was left to settle for 30 min at 30 °C.

### 4.2. Experimental Design

#### 4.2.1. Effect of Heating on Furfural Concentration

Buckwheat honey was diluted with water at room temperature to obtain worts with an extract of 25°Brix (czwórniak), 30°Brix (trójniak) and 45°Brix (dwójniak). The wort was divided into two parts: unacidified (no treatment) and acidified (with added 0.25 g/L of citric acid monohydrate pure p.a. (Avantor Performance Materials, Gliwice, Poland)). A volume of 200 mL of the wort was measured in 500 mL round-bottom flasks and heated to boiling under reflux for various time ranges (from 10 to 70 min) to obtain the saturated worts. The unheated mead wort was used as a negative control (unsaturated wort). The wort samples were then cooled in a stream of cold water and the furfural content was determined using the HS-SPME-GC-MS method. Three replicates of the experiment were performed.

#### 4.2.2. Effect of Furfural Concentration on Mead Fermentation

Buckwheat honey was mixed with potable water in a ratio 1:2 (*v*/*v*), heated and gently boiled for 10 min, then topped up with diammonium hydrogen phosphate(V) (pure p.a., Avantor Performance Materials, Poland; 0.4 g/L) and acidified with citric acid (pure p.a., Avantor Performance Materials, Poland; 0.25 g/L). The wort extract was checked and corrected to 30°Brix after mixing. The hot wort was poured into flasks (0.2 L of wort into 0.5 L flasks) closed with a stopper with sterile fermentation trap tubes filled with glycerin. After cooling the wort to about 20 °C, furfural (Sigma-Aldrich, St. Louis, MO, USA) was added in amounts from 1 to 100 mg/L and a precisely defined amount of starter yeast was added (0.5 g/L calculated on dry substance). Worts without the addition of furfural were treated as control samples.

#### 4.2.3. Control of the Fermentation Process

Fermentation of mead worts with and without furfural was conducted at 20 ± 1 °C and the samples were weighed on a balance PS 4500/X (Radwag company, Radom, Poland), three times per week. The weight loss resulted from the release of carbon dioxide produced during fermentation [45]. The fermentation process was continued until two consecutive weights of the samples, did not differ by more than 1 g. The weight loss was converted into the amount of CO_2_ released in g/100 g. All fermentation experiments were conducted three times.

### 4.3. Analytical Methods

#### 4.3.1. Physicochemical Parameters

Ethanol content, pH and the total and volatile acidity were determined using official methods recommended by O.I.V. [46]. The pH value was measured using a CP-505 pH-meter (Elmetron, Zabrze, Poland). The density of the samples and sample distillates was determined using an oscillating density meter DMA 4500 M (Anton Paar GmbH, Graz, Austria).

#### 4.3.2. Determination of Furfural and Furfural Alcohol Content Using HS-SPME-GC-MS

The analysis was performed using gas chromatography (GC) coupled to time-of-flight mass spectrometry (TOF-MS) after headspace solid-phase microextraction (HS-SPME) [47].

##### Sample Preparation

Headspace solid-phase microextraction (HS-SPME) was performed using a MultiPurpose Autosampler (MPS Dual Head, Gerstel GmbH and Co.KG, Mulheim, Germany). Samples of mead wort and young mead after 7 weeks of fermentation were stored at –20 °C prior to analysis. After heating to 20 °C, 1 mL of the sample was transferred to a 15 mL glass vial, 1 mL of saturated sodium chloride (pure p.a., Avantor Performance Materials, Gliwice, Poland) solution was added and the vial was capped and transferred to the autosampler tray. HS-SPME microextraction (30 min, 40 °C) was performed on PDMS fiber (100 μm, polydimethylsiloxane, SUPELCO, Sigma-Aldrich, St. Louis, MO, USA). Desorption was carried out in the inlet of the gas chromatograph at a temperature of 260 °C.

Calibration curves were prepared in the same way as the analyzed samples. Furfural and furfuryl alcohol (Sigma-Aldrich, St. Louis, MO, USA) were used to prepare calibration solutions.

##### TOF-MS Chromatographic Separation Conditions

The experiments were carried out using an Agilent Technologies gas chromatograph model 7890 B, coupled with time-of-flight mass spectrometer (TOF-MS Pegasus HT, LECO Corporation, St. Joseph, MI, USA). Chromatographic separation was performed on a Restek Stabilwax (cross-linked poly(ethylene glycol) column, dimensions: 30 m; 0.25 mm; 0.25 μm), at the programmed GC column temperature: initial at 35 °C (5 min), ramp 5 °C/min to 110 °C and 40 °C/min up to 230 °C, and final heating for 5 min. SPME fiber desorption was performed in a septumless inlet at a temperature of 260 °C, for 60 s in splitless mode. Helium (6.0, Linde Gaz Polska sp. z o.o., Kraków, Poland) at a flow rate of 1 mL/min was used as the carrier gas. TOF-MS detector parameters: scanning frequency: 20 Hz, acquisition voltage: 1500 V, ionization energy: –70 eV, ion source temperature: 250 °C. The transfer line temperature was 250 °C.

### 4.4. Statistical Analysis

All analytical results were selected for an analysis of variance (ANOVA). The post-hoc analysis of means was performed with Tukey’s test at 5% error probability using Statistica 13.3 software (TIBCO Software Inc., Palo Alto, CA, USA), the graphs were generated using Microsoft Office Professional Plus 2013 software.

## 5. Conclusions

The performed study proved that heating the mead wort to boiling before fermentation correlated with the significantly increased amount of furfural. This should be taken into account when deciding on the length of wort saturation and the method and rate of wort cooling prior to fermentation. Most of the furfural is reduced to furfuryl alcohol during fermentation.

Fermentation of mead worts with 30°Brix extract supplemented with furfural in amounts ranging from 1 to 100 mg/L showed an acceleration of fermentation and an increase in ethanol concentration only in samples with 2 mg/L added furfural. Higher furfural concentrations did not significantly affect the fermentation rate or the basic parameters of the fermented worts. In particular, there was no inhibition of fermentation and no increase in acetic acid synthesis by yeast and volatile acidity, even in samples containing furfural concentrations ten times higher than those naturally occurring in mead worts heated for over one hour. The lack of influence of furfural on the course of fermentation does not mean that the heating of honey worts does not affect the quality of the obtained meads. As a result of an increase in temperature and a decrease in the pH of mead worts, furfural is formed, which, after reduction during fermentation, contributes to an increase in the concentration of furfuryl alcohol in the finished product.

The obtained results can be applied to practical mead production. It should be noted that the heating time is a critical parameter enhancing the furfural production. The boiling time of the mead wort should be restricted, and the solution should be cooled rapidly to minimize Maillard reactions, as these reactions proceed even while the boiled wort cools. Acidification of the wort with citric acid should be performed after cooling the solution. Additionally, it may be beneficial to prepare wort with a higher extract and dilute it after saturation, as this method produces solutions with lower concentrations of furfural and saves the energy required to heat a larger volume of water.

## Figures and Tables

**Table 1:** Parameters of fermented mead worts (30°Brix) with furfural added at concentrations ranging from 1 to 100 mg/L.

		Amount of Furfural Added to the Wort (mg/L)						
		0	1	2	5	10	50	100
Ethanol	(% *v*/*v*)	13.0 ± 0.4 a ^1^	13.4 ± 0.2 ab	14.3 ± 0.3 b	13.7 ± 0.4 ab	13.6 ± 0.2 ab	13.6 ± 0.7 ab	13.6 ± 0.8 ab
Extract	(g/L)	158 ± 3 a	152 ± 8 a	146 ± 5 a	152 ± 2 a	157 ± 1 a	147 ± 13 a	163 ± 9 a
Titratable acidity	(g/L)	3.4 ± 0.1 a	3.4 ± 0.1 a	3.6 ± 0.1 a	3.6 ± 0.1 a	3.7 ± 0.1 a	3.6 ± 0.1 a	3.6 ± 0.1 a
pH		3.32 ± 0.04 a	3.37 ± 0.03 a	3.34 ± 0.03 a	3.46 ± 0.11 a	3.52 ± 0.04 a	3.38 ± 0.01 a	3.38 ± 0.01 a
Volatile acidity	(g/L)	1.3 ± 0.1 a	1.2 ± 0.1 a	1.4 ± 0.1 a	1.4 ± 0.1 a	1.4 ± 0.1 a	1.2 ± 0.2 a	1.3 ± 0.1 a

^1^ Values are expresses as the mean ± standard deviation. Means with different letters (a,b) are statistically different (*p* < 0.05).

## Data Availability

Data are contained within the article.
